# Stochastically Bundled Dissipators for the Quantum Master Equation

**DOI:** 10.1021/acs.jctc.5c00145

**Published:** 2025-04-02

**Authors:** Sayak Adhikari, Roi Baer

**Affiliations:** Fritz Haber Center for Molecular Dynamics and Institute of Chemistry, The Hebrew University of Jerusalem, Jerusalem 9190401, Israel

## Abstract

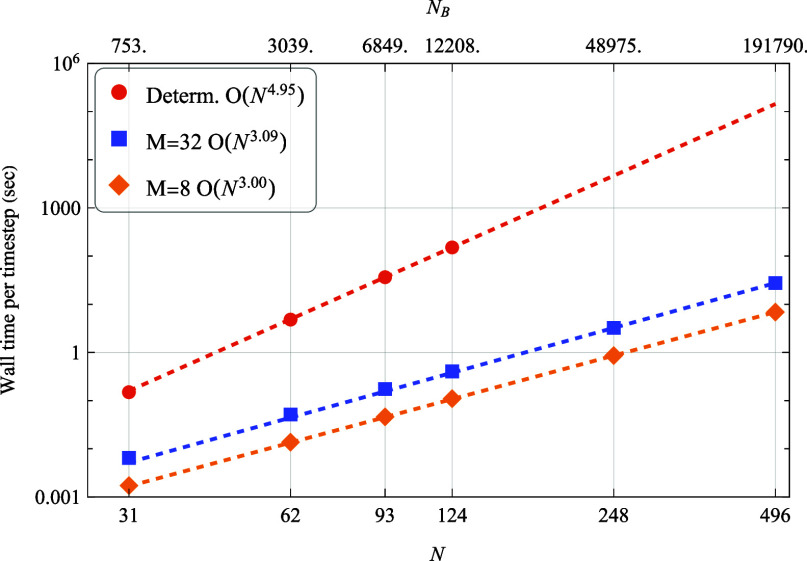

The Lindblad master equation is a fundamental tool for describing the evolution of open quantum systems, but its computational complexity poses a significant challenge, especially for large systems. This article introduces a stochastic representation of the Lindblad dissipator that addresses this challenge by bundling the Lindblad operators. The stochastic dissipator maintains the Lindblad form, ensuring completely positive and trace-preserving dynamics. We demonstrate the effectiveness of this method by considering a Morse oscillator coupled to a spin bath. Our numerical experiments show that a small number of stochastically bundled operators can accurately capture the system’s dynamics, even when the Hilbert space dimension is large. This method offers a new perspective on open quantum systems and provides a computationally efficient way to simulate their dynamics.

## 1 Introduction

Quantum systems, by their nature, interact with their environment, making them inherently “open” to external influences. In many applications, their dynamics can be effectively modeled using Markovian master equations applied to the system’s density matrix.^1−4^ The Lindblad Master Equation (LME)^5−10^ is the fundamental Markovian quantum master equation used to describe this evolution. Independently developed by Lindblad^11^ and Gorini, Kossakowski, and Sudarshan,^12^ the LME is given by:
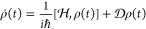
1where  is the effective Hermitian Hamiltonian, and the quantum dissipator  has the form
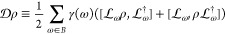
2here, *B* is a set of indices ω, γ(ω) are non-negative coupling functions and the Lindblad operators  capture the effects of the environment on the system. The LME preserves the positivity and unit trace of the reduced density operator, crucial for probability calculations.

In chemical physics researchers often use the LME to study energy transfer, relaxation processes, and chemical reactions.^13−20^ It is also utilized in condensed matter physics to investigate transport phenomena, nonequilibrium dynamics, and the behavior of disordered and strongly correlated systems.^21−25^ Within quantum information science, it plays a crucial role in modeling decoherence in qubits, analyzing quantum gates, and characterizing noisy communication channels.^26−28^ Furthermore, it is essential for advancements in quantum thermodynamics, where it elucidates fundamental limits on energy conversion and information processing.^9,29−31^

Beyond these specific applications, the LME has also fundamentally changed our understanding of open quantum systems. It expresses the idea that the evolution of a pure state is shaped by both the system’s own internal dynamics and its random interactions with the environment.^32,33^ In fact, the Lindblad operators themselves have been interpreted as representing random measurements performed by the environment on the system.^6,34−40^

Stochastic approaches like quantum jump^41−44^ and quantum state diffusion^45,46^ provide a way to sample these random system trajectories over time, and when averaged, these trajectories yield the Lindbladian density matrix.

Davies^47,48^ derived the form of the effective Hamiltonian and Lindblad operators for systems weakly coupled to a thermal environment at temperature *T*, described by the density matrix ρ_*E*_(*T*). In this formalism, the system Hamiltonian is , and the system-environment coupling is , where  and  are system and environment operators, respectively. The set *B* corresponds to the distinct Bohr frequencies ω arising from energy level differences in . The effective Hamiltonian is
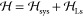
3

with the Lamb-shift operator
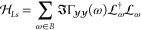
4where
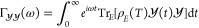
5

is the Fourier transform of the environment operator’s autocorrelation function, and the Lindblad operators are
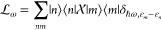
6where ε_*n*_ and |*n*⟩ are, respectively, the eigenvalues and eigenkets of . Davies also gave the explicit form for the coupling function , noting it satisfies the detailed balance condition: , ensuring that the LME drives the system to thermal equilibrium
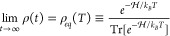
7

as required for systems weakly coupled to the environment.

While the Davies formalism provides a valuable framework for deriving the LME, its practical application to large quantum systems faces significant computational hurdles. Before tackling the computational challenges of applying Lindblad equations to large systems, their validity in this regime must be established. The Davies approach, often used to derive Lindblad operators (eq 6 of the main text), is limited when applied to systems with a high density of states—such as nanocrystals, semiconductors, and light-harvesting complexes—because it relies on the secular approximation, which assumes well-separated energy levels. However, recent developments have yielded alternative methods that transform the Redfield equation into a Lindblad form without requiring the full secular approximation.^49−54^ These methods validate the use of the LME for studying open quantum systems with dense state spaces.

Given the validity of the LME for these systems, we now turn to the computational challenges associated with its numerical solution. These challenges have spurred the development of specialized software packages, libraries, and stochastic algorithms.^55−58^ The computational complexity scales dramatically with the Hilbert space dimension. This scaling arises from the increasing number of Lindblad operators needed to describe the system’s interaction with its environment. Each operator represents a potential pathway for the system to exchange energy or information with the environment, leading to a complex dissipator term in the LME.

To illustrate these challenges, consider a Hilbert space of finite dimension *N*. Evolving the *N* × *N* density matrix ρ via the LME involves operations with matrices of the same dimension representing the Hamiltonian and Lindblad operators. Assuming no special structure or sparsity, and recognizing that the number of Lindblad operators scales as ,^6^ we analyze the complexity of two common approaches. First, consider direct application of the dissipator. Preparing the Lindblad matrices requires  operations. Applying the dissipator to ρ involves  matrix multiplications (one for each Lindblad operator), each with a complexity of , resulting in an overall scaling of  per time step. A second approach utilizes the Lindbladian superoperator, an *N*^2^ × *N*^2^ matrix. Constructing this superoperator is formally  but is performed only once. Subsequent propagation by applying the superoperator to ρ requires  operations per time step.

This high computational cost underscores the need for efficient numerical methods. These challenges can arise even for systems with a relatively small number of degrees of freedom if they are strongly coupled to their environment. In such scenarios, a common strategy is to incorporate relevant environmental modes (e.g., reaction coordinates) into the system Hamiltonian. By renormalizing environmental correlation functions, this approach effectively transforms a strongly coupled small system into a weakly coupled larger system, often leading to more tractable calculations.^59,60^ This transformation further motivates the development of optimized numerical techniques for large systems.

This paper introduces a novel method for addressing the computational bottleneck arising from the quadratic growth in the number of Lindblad operators with increasing system size. We demonstrate that the standard Lindblad dissipator can be transformed into a stochastic dissipator containing a fixed number of “bundled” Lindblad operators, significantly reducing the complexity of the calculations while preserving the essential Lindblad form and maintaining accuracy within a specified error threshold for relevant observables.

## 2 Method

### 2.1 Stochastic Bundled Dissipators

Our procedure involves a vector , whose components are real or complex independent random variables, each with zero expected value and unit variance
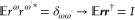
8

For example, *r*^ω^ can be discrete random variables sampling from {−1,1} or a random real number in the interval [−√3,√3]. Another example, used in the test cases shown below, is to take  as a random point on the complex unit circle, where θ_ω_ is a random angle in the interval [0,2π). We then form the stochastically bundled Lindblad operator
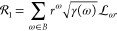


with which we can define the random dissipator as a formal operation
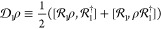
9

The full dissipator defined in eq 2 is the expected value of the bundled dissipator
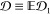
10

The fluctuations in  can be mitigated by statistical sampling, using *M* > 1 random vectors *r*_*m*_, *m* = 1, ..., *M*, generating the bundled operators, 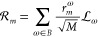
11

from which we define a stochastically bundled dissipator of size *M*
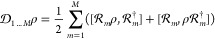
12

The full dissipator is still the expected value of the random dissipator:  but  has a smaller fluctuation, by a factor *M*^–1/2^, than . Notably, both  and  maintain the Lindblad form, ensuring a completely positive and trace-preserving evolution of the density matrix.

Once we have sampled the dissipator  we use it in the following stochastic LME
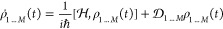
13

to evolve the density matrix from its known initial value to all time values. The evolving ρ_1···*M*_(*t*) is now a random density matrix with which we can estimate the exact deterministic ρ(*t*) obtained from the LME with the full dissipator (eq. 1).

### 2.2 Bias Mitigation via Jackknife Resampling

The stochastic fluctuations in , which are proportional to *M*^–1/2^ will cause the expected values of ρ_1···*M*_(*t*) to differ from ρ(*t*), resulting in a “bias”-ed estimator
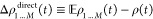
14where the superscript direct emphasizes the direct use of ρ_1···*M*_(*t*) in the estimation of ρ(*t*). The bias occurs because the noise in the bundled dissipator enters the evolving density matrix in a nonlinear way.

The bias typically drops as *M*^–1^ for large sample sizes *M*(61) and we can reduce the bias further by combining results from subsets of the bundled operators. This suite of techniques are jackknife estimators and they hold potential to reduce the bias faster than *M*^–1^.^62^ For example, taking an even sample size *M*, the “jackknife_1_” estimator is
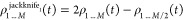
15where ρ_1···*M*_ was defined above and ρ_1···*M*/2_ is the estimator obtained from the stochastic LME using the dissipator , based on . The required numerical work in applying jackknife_1_ is  times that of the direct method. This is because we need to solve the LME twice, once with the  dissipator and once with the  dissipator and then apply eq 15. A more elaborate yet balanced approach is the “jackknife_2_” estimator
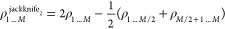
16

The required numerical work in applying jackknife_2_ is 2 times that of the direct method. This is because we need to solve the LME twice, once with the  dissipator and once with the  dissipator and then apply eq 15.

## 3 Results

### 3.1 Anharmonic Oscilator-Spin System

In order to benchmark the stochastic bundling method, we construct a hierarchy of systems where the number of Lindblad operators increases significantly, but the underlying dynamics remain largely unaffected. This approach allows us to readily identify any divergence of the bundling method with growing Hilbert space size, as other system properties are kept relatively constant. Specifically, we consider an anharmonic oscillator coupled to a qudit: spin *s* = 0 (spinless),  (qubit), *s* = 1 (quit or photon), and  (quart).

#### 3.1.1 The Hamiltonian

The Hamiltonian describes a spin *s* coupled to a Morse oscillator
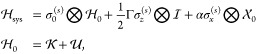
17where Γ is the spin gap parameter, α is the spin-oscillator coupling constant, and the oscillator Hamiltonian  includes kinetic energy  and potential energy . Here,  is the oscillator position operator, and the potential is defined as
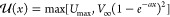
18

Further details regarding the model are provided in the Methods section.

We assume that the system operator coupling to the environment is the oscillator position . Neglecting the Lamb shift, we model the coupling function γ(ω) as
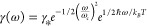
19with ω_*c*_ being a high-frequency cutoff (*k*_*B*_ and *ℏ* are Boltzmann’s and Planck’s constants, respectively; see Supporting Information for values of other parameters).

The Hamiltonian corresponding to spin *s* are given in eq 17 where the (2*s* + 1)×(2*s* + 1) matrices are:

- σ_0_^(*s*)^ = 1_2*s*+1_ is the (2*s* + 1)-dimensional unit matrix
- σ_*z*_^(*s*)^ is the diagonal matrix with entries  on the main diagonal
- σ_*x*_^(s)^ is a tridiagonal matrix with 0s on the main diagonal and 1’s on the sub- and superdiagonals.

The Morse oscillator, a Hilbert space consisting of continuous wave functions ψ(*x*) supported on the interval *x*∈[*x*_0_,*x*_*f*_] (vanishing outside this interval). We discretize these functions, representing them as *N*_*x*_ + 1-dimensional vectors ψ_*n*_ ≡ ψ(*x*_*n*_) where
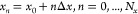
20are internal grid points. The function is assumed zero for *n* < 0 and *n* > *N*_*x*_. (see Table 1 for values of the various parameters).

**Table 1:** Parameters of the Calculation (We use Atomic Units)

*m*	1	oscillator mass
[*x*_0_,*x*_*f*_]	[−10,20]	real space grid parameters eq 20
*N*_*x*_	30	
Δ*x*	1	
*V*_*∞*_	4	morse potential parameters, eq 18
*U*_max_	6	
*a*	0.2	
ω_*c*_	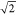	coupling function parameters, eq 19
γ_*_	0.005/0.02	
*k*_*B*_*T*	0.25/1	
ξ	3.4/0.7	initial state, eq 23
δ*t*	0.25/0.125	Runge–Kutta time step. The larger γ_*_ the smaller δ*t*
Γ	0.1	spin gap and oscillator-spin coupling eq 17
α	0.1225	

The total dimension of the oscilator-spin Hilbert space is (*N*_*x*_+1)(2*s* + 1). Since *N*_*x*_ = 30, we have 31 dimensions for *s* = 0, 62 for , 93 for *s* = 1, etc.

The position operator  and potential  act as follows:  and  respectively, where *U*(*x*) is the Morse potential given in eq 18.

The kinetic energy operator , is approximated using a sixth-order (seven-point) finite-difference approximation for the second derivative
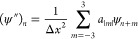
21where
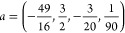
22

combined with the boundary condition  for all positive *k*.

**Figure 1:**
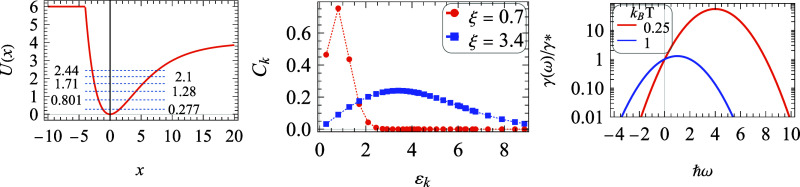
Left: the Morse potential *U*(*x*) used in this example. The dashed lines indicate the six low-lying eigenvalues (the total number of eigenvalues is 31). Middle: the coefficients *C*_*k*_ for the *k*th eigenstate of energy ε_*k*_ of the cold and hot initial states of the spinless system (see eq. 23). Right: The coupling function γ(ω) (eq. 19) for the two temperatures used in this study.

The potential and lowest-lying energy levels of the oscillator’s Hamiltonian are shown in the left panel of Figure 1.

#### 3.1.2 The Initial States

We take the initial density matrix as a pure state , where
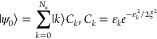
23where |*k*⟩ and ε_*k*_ are the eigenstates and eigenvalues of the total Hamiltonian and the parameter ξ takes two values: 0.7 (“cold”), and 3.4 (“hot”). The coefficients *C*_*k*_ corresponding to these values are shown in the middle panel of Figure 1.

#### 3.1.3 The Lindblad Equation Propagator

The fourth-order Runge–Kutta propagator with a time step of δ*t* (see Table 1) is used to solve the Lindblad Master Equations starting from the initial pure state, obtaining
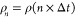
24where Δ*t* = 1*ℏ*/*E*_*h*_ and *n* = 1, 2, ... . We record the energy , position  and purity  transients. The parameter δ*t* is small enough to bring all solutions to convergence of 9 significant digits.

### 3.2 The Cooling and Heating Dynamics

**Figure 2:**
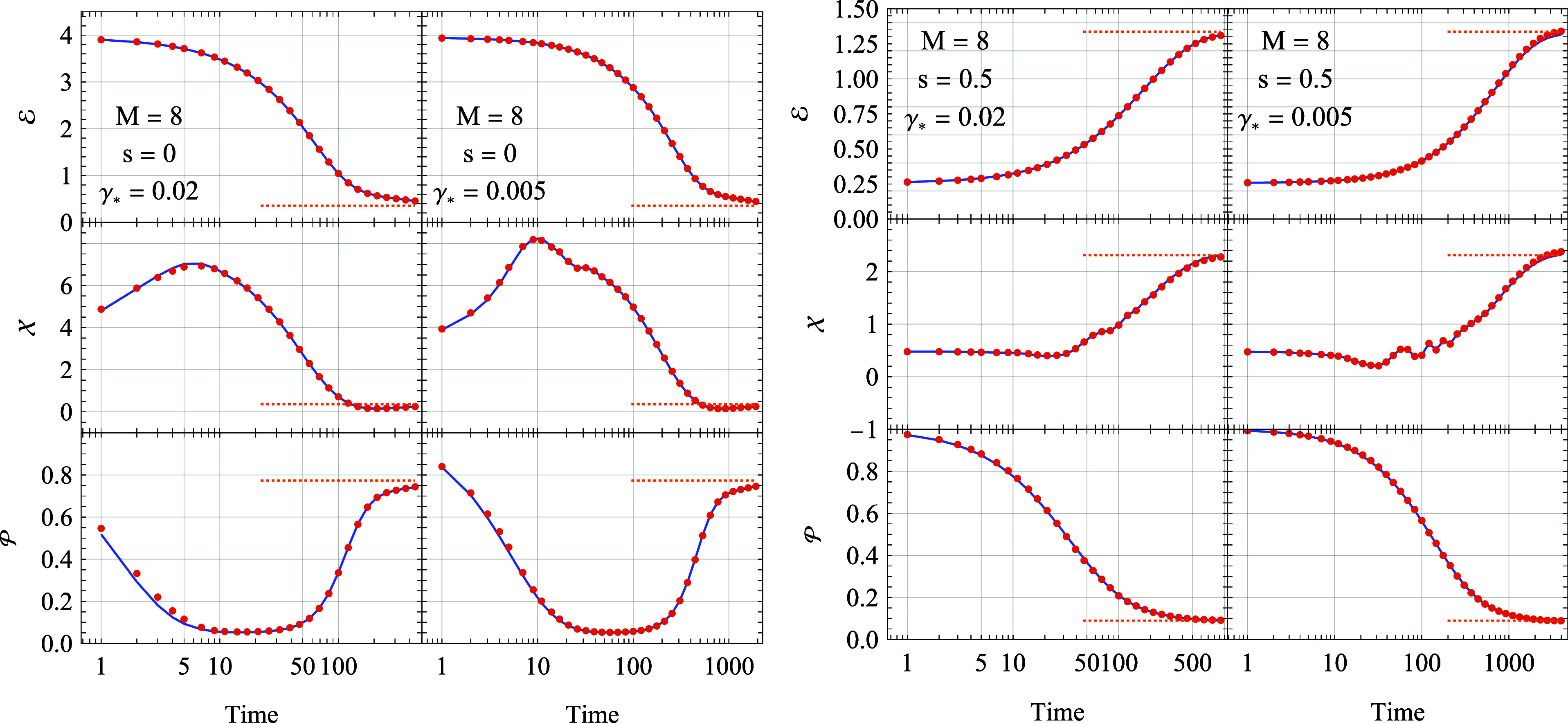
Energy , position , and purity  transients for a Morse oscillator initially in a pure state (eq. 23). Left panels: oscillator without spin (*s* = 0) undergoing cooling (ξ = 3.4, *k*_*B*_*T* = 0.25). Right panels: oscillator coupled to a spin  experiencing heating (ξ = 0.7, *k*_*B*_*T* = 1*).* Deterministic (solid lines) and stochastic (markers) dynamics are compared. Dashed lines indicate thermal equilibrium values. The deterministic dynamics are based on 753 (left panels) and 3287 (right panels) Lindblad operators. The stochastic dynamics in both panels use *M* = 8 bundled operators. The error bars for the stochastic estimates are consistently smaller than the marker size (see Figure 3). An in-depth error analysis is provided below.

Figure 2 illustrates the dynamics of our system under two distinct scenarios and coupling regimes:

Cooling Scenario (Figure 2, left panels): a hot, spinless oscillator (ξ = 3.4) is coupled to a low-temperature bath (*k*_*B*_*T* = 0.25) with environmental coupling parameters γ_*_ = 0.02 and γ = 0.005. The energy  decays monotonically toward its equilibrium value, exhibiting faster relaxation in the strong coupling regime. The oscillator position  initially expands rapidly from 4 to approximately 8 (strong coupling) or 9 (weak coupling) before slowly contracting to its equilibrium value of . The purity experiences a rapid initial drop from unity, followed by a prolonged period at , finally increasing toward the thermal equilibrium value of  as the system approaches its ground state.

Heating Scenario (Figure 2, right panels): a cool (ξ = 0.7) oscillator coupled to a spin- is immersed in a hot bath (*k*_*B*_*T* = 1). The energy increases monotonically, albeit with slower timescales compared to the cooling scenario. In the weak coupling regime, the position exhibits coherent oscillations at intermediate times (*t* ≈ 100) as it approaches its thermal equilibrium length. The purity decays monotonically toward its equilibrium value of .

### 3.3 The Stochastic Calculation

The stochastic approach estimates the expectation value of an observable  at time *t* as , where ρ_1···*M*_(*t*) is the solution at time *t* of the LME with a stochastic dissipator (Eq. 13) having *M* bundled Lindblad operators. Figure 2 shows the expectation value estimates of the energy, position, and purity based on a single *M* = 8 stochastic calculation (a single run, no statistical averaging). Despite the fact that we do not rerun the calculation many times, the obtained estimates closely approximate those of the full deterministic calculation, which utilizes a dissipator with over 725 (spinless oscillator) and 3287 (oscillator coupled to spin ) Lindblad operators, despite being approximately 2 orders of magnitude faster to compute for the spinless case, and nearly 400 times faster for the case with spin.

**Figure 3:**
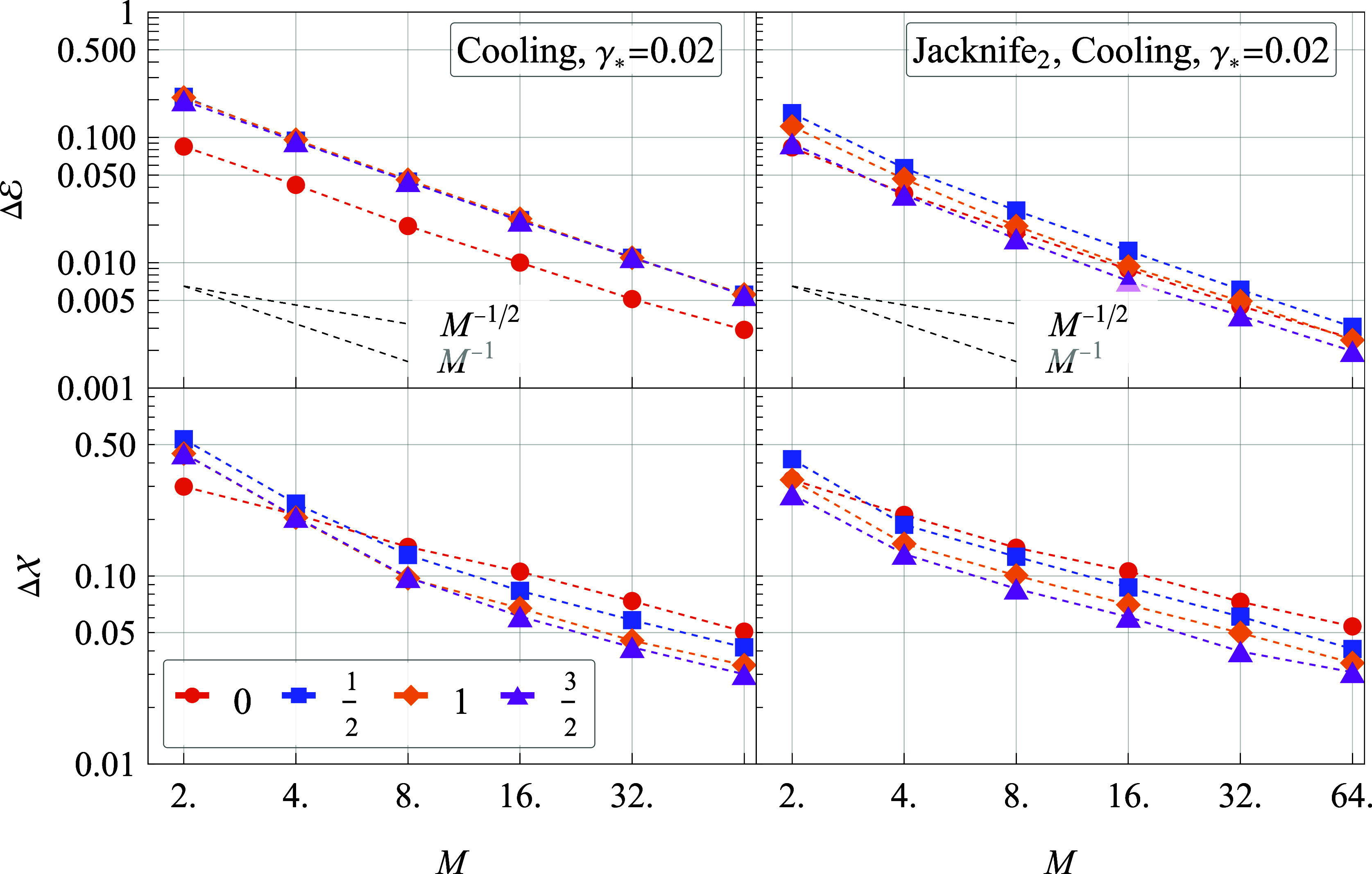
Maximal RMSEs in energy and position for open spin-oscillators coupled to a cold environment (*k*_*B*_*T* = 0.25) shown as functions of the number of stochastically bundled Lindblad operators, M. Calculations start from an initially pure and hot state. Dashed lines indicate M^–1^ and M^–1^/^2^ slopes.

The computational speedup achieved through the stochastic approach comes at the cost of reduced accuracy. To quantify this, we examine the maximum root-mean-square error (RMSE) for energy, , and position, , in the cooling scenario with coupling strength γ_*_ = 0.02, as depicted in Figure 3. The definition of the RMSE and the methods used for its determination are detailed in the Supporting Information. Results for the other scenarios exhibit similar trends and are presented in the Supporting Information.

For energy, the lowest  values occur for the spinless system. Increasing the spin to , where the Hilbert space dimension increases by a factor of 2 and the number of Bohr frequencies by a factor of 4,  grows by a factor of 2.5. Further increases to *s* = 1 and , doubling once more the Hilbert space dimension, do not significantly change  further. The maximal RMSE in position, , exhibits different behavior:  decreases with increasing spin and Hilbert space dimension. We conclude that consistently increasing Hilbert space dimensions does not significantly affect the statistical errors. Therefore, the number *M* of stochastically bundled Lindblad operators can remain constant as the Hilbert space dimension *N* grows, without sacrificing accuracy.

Next, we discuss the dependence of the maximal RMSEs on *M*. While  decreases proportionally to *M*^–1^,  behaves differently. For nonzero spin and *M* ≤ 8,  decreases as *M*^–1^, and in other cases (*M* > 8) it decreases as *M*^–1/2^.

Analysis of the mean squared error (MSE) provides insight into the observed behavior. The MSE is decomposable into the sum of the variance and the squared bias, with definitions and estimation procedures for these components detailed in the Methods section.

Statistical theory establishes that both bias and variance exhibit scaling relationships with sample size (*M*), scaling as *M*^–1^.^63^ Consequently, when the MSE is dominated by variance, its root, the root mean squared error (RMSE), will be approximately equal to the standard deviation, thus scaling proportionally to *M*^–1/2^. Conversely, when the MSE is dominated by the squared bias, the RMSE approximates the bias, scaling as *M*^–1^.

Therefore, the dominant error source within the MSE can be inferred from the RMSE scaling behavior: *M*^–1^ scaling indicates bias dominance, whereas *M*^–1/2^ scaling signifies variance dominance. This relationship is further corroborated by the effect of the jackknife resampling method on the maximal RMSE, as illustrated in Figure 3. When bias dominates, the jackknife effectively reduces the maximal RMSE. In contrast, when variance dominates, the jackknife exerts a negligible effect on the maximal RMSE.

**Figure 4:**
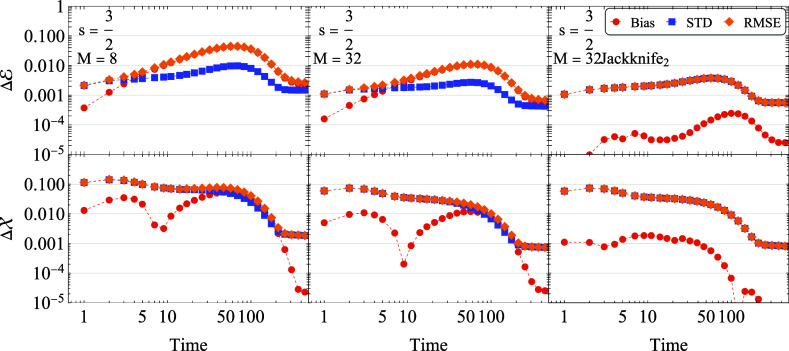
Errors in energy (top) and position (bottom) for the cooling scenario of a spin-3/2 oscillator system with γ_*_ = 0.02. The left panels show errors for a bundled dissipator with 8 Lindblad operators, the middle panels for 32 operators, and the right panels for 32 operators within the jackknife approach of eq 16.

**Figure 5:**
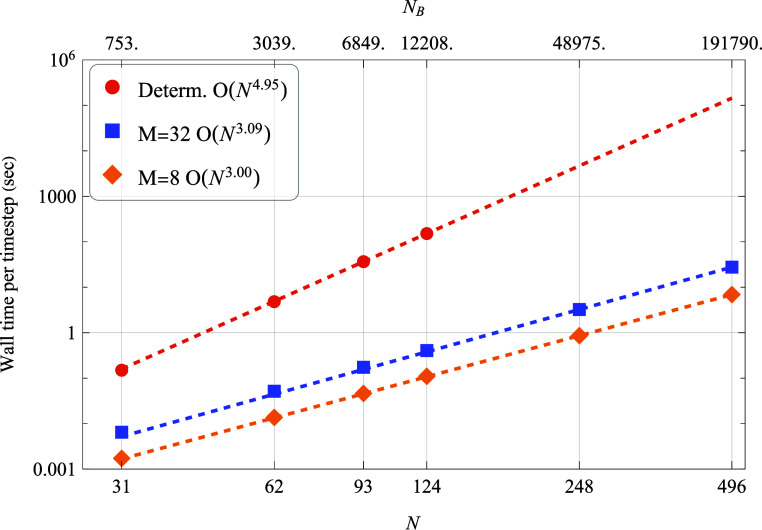
Wall times for oscillator-spin systems as function of the Hilbert space dimension *N* (and the corresponding number of Bohr frequencies, *N*_*B*_). The scaling of the calculations, *n*, is determined by fitting the data to *t* = *aN*^*n*^.

To demonstrate this effect further, Figure 4 presents the time-dependent RMSE, fluctuations (standard deviation), and bias for energy and position observables at , with results for *M* = 8 (left) and *M* = 32 (right). For the energy observable, the bias error generally dominates the RMSE, particularly at the maximal RMSE occurring around *t* = 50. This observation confirms the *M*^–1^ scaling of the maximal RMSE for energy. In contrast, the maximal RMSE for the position observable , observed at early times, is dominated by fluctuation error, consistent with the expected *M*^–1/2^ scaling.As previously discussed, solving the Lindblad equation has a formal algorithmic complexity of  per time step, where *N* is the Hilbert space dimension. Our results demonstrate that the number *M* of bundled Lindblad operators is largely independent of *N*. This independence implies a significant reduction in complexity to . This improved scaling is confirmed by our calculations, as shown in Figure 5. The  scaling of deterministic calculations quickly becomes computationally prohibitive. In contrast, our stochastic approach can handle systems described by hundreds of thousands of Lindblad operators in the traditional formalism, effectively reducing the computational cost.

## 4 Discussion

The stochastic bundling method, introduced in this paper, offers a powerful new approach to calculating the dissipator in the Lindblad master equation, a key challenge in the study of open quantum systems. By approximating the dissipator with a reduced set of stochastically bundled Lindblad operators, the method significantly lowers computational complexity while preserving the essential Lindblad form, ensuring completely positive and trace-preserving dynamics.

Our numerical experiments, including the example of a Morse oscillator coupled to a spin-1/2 particle, demonstrate the effectiveness of this method. We were able to closely approximate the system’s dynamics using only *M* = 8 bundled operators instead of the full set of 3069 Lindblad operators, resulting in a 400-fold reduction in computation time. This highlights the potential of stochastic bundling to address the computational bottleneck associated with large open quantum systems.

While significantly reducing computational cost, it is crucial to assess the accuracy of the stochastic bundling method. The accuracy is influenced by factors such as the specific system under investigation, the desired accuracy, the number of bundled operators employed, and the application of jackknife resampling. Notably, our findings demonstrate that the number *M* of bundled operators required to attain a given accuracy level does not scale with the Hilbert space dimension, *N*. This results in a time propagation scaling of  for our method, a substantial improvement over the  scaling of traditional deterministic approaches (where *N*_*t*_ is the number of time steps). This scaling advantage, along with a reduction in memory from  to , renders stochastic bundling highly effective for large systems with high-dimensional Hilbert spaces. It is important to acknowledge that the preparation of Lindblad operators, irrespective of bundling, scales as .

We have also demonstrated the effectiveness of jackknife resampling in mitigating bias. This is crucial for interpreting our method as a physically meaningful approximation. When the bias, introduced by using a finite number of bundled operators, is sufficiently small, the random density matrix ρ_1···*M*_(*t*) (obtained from Eq. 13) can be interpreted as representing a plausible trajectory of the system’s mixed state under environmental fluctuations. While fluctuation error remains it can be mitigated through repeated calculations and averaging, as the fluctuations tend to cancel out.

This approach bears resemblance to unraveling via quantum state diffusion (QSD) processes^45,64^ and the quantum trajectories methods (QTM),^41,43^ which involve solving a stochastic Schrödinger equation (SSE) that yields evolving pure states. Each of these pure states represents a plausible evolution of the system under the influence of a fluctuating environment, and averaging over them produces the exact density matrix ρ(*t*). Crucially, our unraveling differs from QSD in that our random states are inherently mixed. Furthermore, these mixed states exhibit significantly smaller fluctuations compared to the pure states in QSD. This difference suggests that our method may offer advantages in terms of stability and efficiency for certain systems, potentially due to the reduced sensitivity to numerical errors arising from the smaller fluctuations.

The bundled dissipators can also be used directly within the QSD and QTM calculations, with the bundled operators acting as jump operators. In future studies, it will be interesting to investigate whether this can improve the performance of QSD and QTM methods. In a recent study,^58^ we developed a second-order approach to solving the SSE for QSD. Its drawback was the dependence on the square of the number of Lindblad operators in the dissipator. It would now be valuable to explore how the reduced number of Lindblad operators (*M*) in the bundled dissipator can expedite the QSD calculations in this case.

Our results demonstrate that explicitly representing every non-negligible Lindblad operator  is not required. Instead, these operators can be effectively bundled into a smaller set of  operators, with the resulting Lindblad dissipator still providing reasonable accurate approximations to the dynamics. This suggests that dissipative Lindblad dynamics may possess a simpler underlying structure than previously assumed.

The  scaling of Lindblad operators can limit the scope of open quantum systems explored in the literature potentially biasing research toward systems with symmetries or locality constraints that reduce operator complexity. While symmetries can simplify the problem by reducing the number of significant coupling constants (e.g., a harmonic oscillator linearly coupled to a harmonic bath requires only one Lindblad operator), many realistic systems do not exhibit such idealizations. This work overcomes the challenges associated with this scaling, enabling the investigation of previously inaccessible systems, such as anharmonic systems, without relying on simplifying assumptions.

While the stochastic bundling method offers significant advantages for studying open quantum systems, it also has limitations that suggest avenues for future research. Currently, the method assumes a weak coupling between the system and its environment. As the coupling strength increases, a larger number of bundled operators, M, may be required to maintain accuracy. Investigating the method’s performance in the strong-coupling regime is therefore crucial. One potential approach is to employ the reaction coordinate method,^59,60^ which effectively maps a strongly coupled system to a larger, weakly coupled one. Another limitation is the method’s restriction to Markovian systems, which lack memory effects. Extending the stochastic bundling technique to non-Markovian systems presents a significant challenge, primarily because the dissipator’s definition becomes considerably more complex, and the applicability of bundling is unclear. Future work should explore the possibility of extending this approach to non-Markovian systems, potentially including those described by Redfield equations.

Despite these limitations, the stochastic bundling method presents a valuable new tool for understanding open quantum systems, especially those with high dimensionality. This dramatic improvement in computational efficiency, stemming from the reduced scaling, enables the simulation of much larger and more complex quantum systems than previously feasible. The ability to explore these systems opens up new avenues for investigating a wide range of phenomena in quantum optics, condensed matter physics, and quantum information science. The stochastic bundling method, therefore, not only addresses a critical computational bottleneck but also expands the scope of quantum systems amenable to theoretical investigation.

## Supporting Information Available

Details for the statistical analysis. Additional numerical snapshots and data concerning the efficacy of the jackknife resampling for bias mitigation (PDF)
