# Supplementary material for: Stochastically Bundled Dissipators for the Quantum Master Equation
Source: J Chem Theory Comput. 2025 Apr 2;21(8):4142–50. doi: 10.1021/acs.jctc.5c00145 (PMC12020372; doi:10.1021/acs.jctc.5c00145)
Supplement: Supplementary file 1 — ct5c00145_si_001.pdf [file ct5c00145_si_001.pdf]

Supplementary Information for:

Stochastically bundled dissipators for the quantum master equation

Sayak Adhikari and Roi Baer*

Fritz Haber Center for Molecular Dynamics and Institute of Chemistry, The Hebrew University of Jerusalem, Jerusalem 9190401, Israel

This supplementary information provides additional snapshots of stochastic transients and information about the jackknife errors.

S-1 Details for the statistical analysis

S-1.1 Our stochastic estimates and their errors

For a given full dissipator $\mathcal{D}$, suppose we solve numerically to high precision the LME of Eq. 1 of the main text obtaining $\rho(t)$, the time-dependent density matrix. Let $\mathcal{O}$ be some observable of interest with the expectation value

$$\langle\mathcal{O}\rangle_t \equiv \mathrm{Tr}\left[\rho(t)\,\mathcal{O}\right]$$

at time t. $\rho(t)$ and $\langle\mathcal{O}\rangle_t$ are the "exact" values. Our method is stochastic, so it does not yield the exact values. It provides an estimate. The bundled LME of Eq. 13 of the main

text allows us to obtain a random density $\rho_{1\ldots M}(t)$, and with it comes *our estimate* for $\langle\mathcal{O}\rangle_t$, which is the following random variable:

$$\langle\mathcal{O}_{1\ldots M}\rangle_t \equiv \mathrm{Tr}\left[\rho_{1\ldots M}(t)\,\mathcal{O}\right].$$

How "good" is this estimate? In other words, what is the mean square error

$$\mathrm{MSE}\left[\mathcal{O}_{1\ldots M}(t)\right] \equiv \mathbb{E}\left(\langle\mathcal{O}\rangle_t - \langle\mathcal{O}_{1\ldots M}\rangle_t\right)^2?$$

This is a standard problem in parameter estimation theory, where the MSE

$$\mathrm{MSE}\left[\mathcal{O}_{1\ldots M}(t)\right] = \mathrm{STD}_t^2 + \left|\Delta O_{1\ldots M}\right|^2, \tag{S-1}$$

is a sum of the variance

$$\mathrm{STD}_t^2 \equiv \mathbb{E}\left[\left(\langle\mathcal{O}_{1\ldots M}\rangle_t - \mathbb{E}\,\langle\mathcal{O}_{1\ldots M}\rangle_t\right)^2\right] \tag{S-2}$$

and the square of the bias:

$$\Delta O_{1\ldots M}(t) = \mathbb{E}\,\langle\mathcal{O}_{1\ldots M}\rangle_t - \langle\mathcal{O}\rangle_t\,. \tag{S-3}$$

S-1.2 Estimating the mean square error and its components

To estimate the MSE, we repeatedly solve the bundled LME of Eq. 13 of the main text N_{runs} times, where $N_{\mathrm{runs}} \gg 1$, each time a independent dissipator is randomly generated. Each run produces a sample $\langle\mathcal{O}_{1\ldots M}\rangle_t^{(n)}$, where $n = 1, \ldots, N_{\mathrm{runs}}$. From this set of samples, we can estimate the expected value $\mathbb{E}\langle\mathcal{O}_{1\ldots M}\rangle_t$ and the variance STD_t^2 used in the MSE calculation (Eq. S-1-S-3). We use the sample average (A_t) and sample standard deviation squared (S_t^2)

as estimators, as described in[63]. The sample average is defined as

$$A_t = \frac{1}{N_{\rm runs}} \sum_{n=1}^{N_{\rm runs}} \langle \mathcal{O}_{1\ldots M} \rangle_t^{(n)},$$

and the sample variance is given by

$$S_t^2 = \frac{1}{N_{\rm runs}} \sum_{n=1}^{N_{\rm runs}} \left(\langle \mathcal{O}_{1\ldots M} \rangle_t^{(n)} - A_t \right)^2 .$$

A_t serves as a good estimator of the true expected value $\mathbb{E}\langle \mathcal{O}_{1\ldots M} \rangle_t$, with fluctuations of approximately $S_t/\sqrt{N_{\rm runs}}$. The difference $A_t - \langle \mathcal{O} \rangle_t$ is an estimator of the bias $\Delta \mathcal{O}_{1\ldots M}(t)$, exhibiting fluctuations of the same magnitude as A_t (i.e., $S_t/\sqrt{N_{\rm runs}}$). Furthermore, S_t is a good estimator of the variance STD_t^2. In summary, we have the following estimates: Bias $\Delta O_{1\ldots M}(t) \approx A_t - \langle \mathcal{O}_{1\ldots M} \rangle_t$, and Standard Deviation $\text{STD}_t \approx S_t$. To ensure the accuracy of the bias estimate, the fluctuations in A_t must be significantly smaller than the bias itself. Consequently, $N_{\rm runs}$ must be sufficiently large to satisfy the condition $\frac{S_t}{\sqrt{N_{\rm runs}}} \ll |\Delta O_{1\ldots M}(t)|$.

S-2 Observable transients as a function of spin

When the spin changes by jumps of $\frac{1}{2}$ from $s = 0$ to $s = \frac{3}{2}$ the number of states changes from 31 to 124. The number of Lindblad operators is the square of these values, so it changes from about 900 to about 14000. The observable transients shown in Figure S-1 are not very sensitive to the spin. This makes our check of stochastic errors ideal, since if there was a sensitivity of the fluctuations or bias to the size of the Lindblad manifold (going from more than 735 to more than 12000) we would easily identify it.

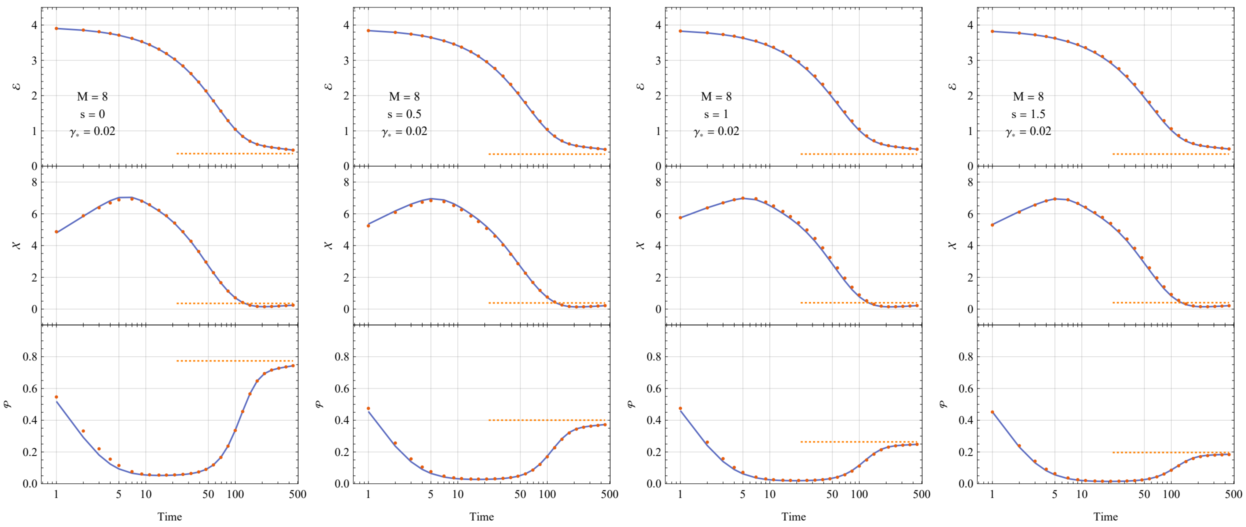

Figure S-1: Additional transients as a function of spin (Hilbert space dimension). As the spin grows the purity drops, the energy and position transients do not change much.

S-3 Observable transients for varying number of bundles with/out jackknife resampling

In Figure S-2 we show the same transients calculated with small number of stochastic Lindblad operators ($M = 2$, 4, and 8), without and with jackknife resampling. Even $M = 2$ gives a very useful transient. The jackknife resampling visibly improves results for $M \geq 4$.

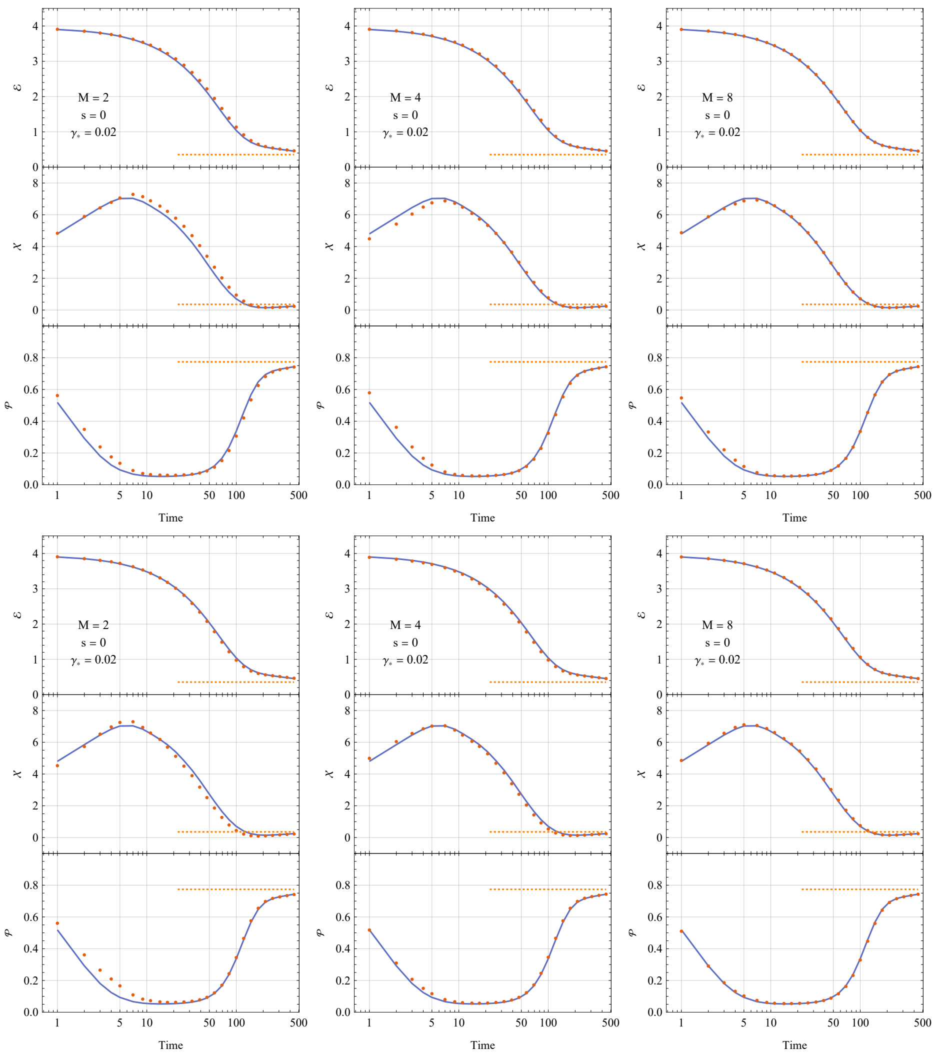

Figure S-2: Description of the transients with increasing M. The bottom row includes jackknife resampling.

S-4 Efficacy of Jackknife resampling for bias mitigation

Figure S-3 illustrates the energy and position error transients for cooling (with environment coupling $\gamma_* = 0.02$) of the various spin-oscillator systems. The number of underlying Lindblad operators increases from 753 for the bare (spin-0) oscillator to 12208 for the oscillator-spin-$\frac{3}{2}$ system. Despite this increase in complexity, the maximum bias and fluctuations do not exhibit a corresponding growth with system size. The efficacy of the Jackknife resampling seems intact as well.

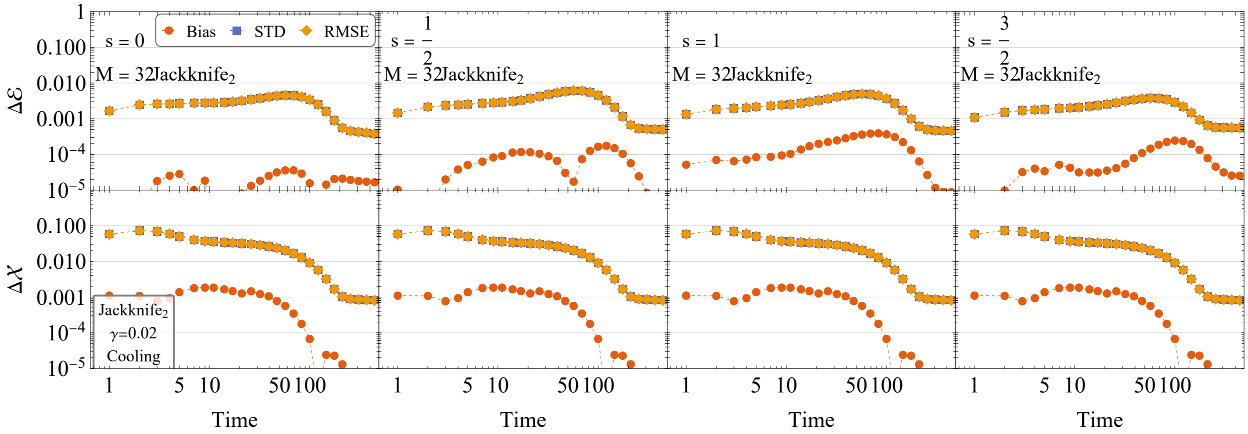

Figure S-3: The dependence of jackknife bias and STD on system size.
